# Clinical Value of TXNDC12 Combined With IDH and 1p19q as Biomarkers for Prognosis of Glioma

**DOI:** 10.3389/pore.2021.1609825

**Published:** 2021-09-22

**Authors:** Xinzhuang Wang, Quan Yang, Nan Liu, Qilong Bian, Ming Gao, Xu Hou

**Affiliations:** ^1^ Department of Neurosurgery, First Affiliated Hospital, Zhengzhou University, Zhengzhou, China; ^2^ Department of Neurosurgery, The First Affiliated Hospital of Harbin Medical University, Harbin, China; ^3^ Department of Neurosurgery, Heze Municipal Hospital, Heze, China

**Keywords:** glioma, prognosis, TXNDC12, IDH, 1p19q

## Abstract

**Background:** Glioma is the primary malignant tumor of the central nervous system and presents high mortality and disability rates under existing treatment measures. Thioredoxin domain-containing 12 (TXNDC12) has been shown to play an important role in various malignant tumors. Therefore, we explored the clinicopathological characteristics of TXNDC12 in glioma to bring to light new ideas in its treatment.

**Methods:** We obtained data packages related to TXNDC12 expression status in gliomas from public databases. We analyzed glioma TXNDC12 expression and patient survival status and validated the above results using glioma specimens from our institution. Next, we analyzed the value of TXNDC12 in combination with 1p19q and isocitrate dehydrogenase (IDH) on the prognosis of glioma by regression model and receiver operating characteristic curve (ROC). Finally, we explored the function of related genes by GO analysis and KEGG analysis.

**Results:** Compared with normal brain tissue, the expression of TXNDC12 in glioma cells, regarding both mRNA and protein levels, was significantly upregulated. The survival time of patients with high-expression of TXNDC12 in glioma cells was shortened. In the World Health Organization pathological classification, IDH status, 1p19q status, and IDH combined with 1p19q subgroups, the expression of TXNDC12 increased with the deterioration of the above indicators. Tumor local immune analysis showed that the immune cell infiltration in TXNDC12 high-expressing glioma tissue increased, the tumor purity was reduced. GO and KEGG analyses indicated that TXNDC12 may be involved in the malignant prognosis of glioma through glycosylation and antigen processing and presentation.

**Conclusion:** We showed that TXNDC12 is significantly highly expressed in gliomas. This high expression predicts the poor prognosis of glioma patients and is related to the gliomas’ local immune microenvironment. As a tumor-related gene, TXNDC12 may be used as a new prognostic judgment molecule.

## Introduction

Gliomas account for 48.6% of all central nervous system (CNS) tumors and 81% of all primary CNS malignancies[1]. Even with the current standard of care, the median survival of newly diagnosed glioblastoma patients is only 12–15 months[2,10], while the pathological progression of low-grade glioma is highly variable[3,4]. Eventually, most low-grade gliomas gradually evolve into high-grade gliomas[5]. It is therefore apparent that the course of different grades of gliomas varies greatly[6], and accurately distinguishing between them is particularly important. The discovery of molecular changes such as 1p19q and IDH has brought major innovations to the diagnosis, treatment, and prognosis of glioma. Carrying IDH mutations or 1p19q codeletion means that patients have a better prognosis[7,10]. However, IDH, and 1p19q are mostly found in grade II and III gliomas, and rarely in grade IV gliomas[8,9], which are the most malignant. In addition, a variety of molecular and pathological pathways are involved in the formation and development of glioma[10,11]. The existing molecular targets have limited relevance to understanding the overall glioma pathogenesis and for guiding treatment. Therefore, it is necessary to develop new molecular markers to improve the classification criteria of glioma.

TXNDC12 is a member of the family of thioredoxin domains, currently known by other names, including ERp16, ERp18, ERp19, AGR1, and hTLP19[12–14]. TXNDC12 is ubiquitously expressed in all tissues and encodes a protein that is primarily involved in catalyzing the formation of disulfide bonds, showing similarities to protein–disulfide bond isomerase activity and playing an important role in endoplasmic reticulum stress[12]. In recent years, researchers have found that TXNDC12 mRNA also plays an important role in tumorigenesis and development[15–17]. For example, the expression level of TXNDC12 mRNA in gastric, liver, and other tumor tissues is significantly higher than that in non-tumor tissues and is significantly associated with poor prognosis of patients[18,19]. TXNDC12 can promote hepatocellular carcinoma metastasis and invasion by upregulating the ZEB1-mediated epithelial-mesenchymal transition process and significantly shortening the postoperative survival of gastric cancer patients[20]. As another member of the thioredoxin family, TXNDC9 is upregulated in gliomas. Knockouts of the TXNDC9 gene can inhibit the proliferation and metastasis of glioma cells and induce apoptosis of glioma cells[21]. Therefore, we speculate that TXNDC12 may be involved in glioma development and may be used as a potential prognostic molecular marker.

Using multicenter and large-sample data, we demonstrated that TXNDC12 is highly expressed in glioma. Its overexpression closely correlates with glioma prognosis, tumor local immune status, IDH status, and 1p19q status, indicating that TXNDC12 may be a promising prognostic marker for glioma.

## Material and Methods

### Obtainment of Clinical Samples

This study was approved by the Ethics Committee of the First Affiliated Hospital of Harbin Medical University (No. HMUIRB-2008-06), and the patients’ consent was obtained. All glioma tissue specimens were taken from surgically resected tissues of patients with glioma (*n* = 25), and non-tumor brain tissue (From patients undergoing epilepsy surgery) was used as a negative control group (*n* = 5). Tissue samples were stored in liquid nitrogen.

### Download and Filtering of the Data Collection

After screening out samples with defects in clinical information, we collected 546 glioma samples and five normal brain samples from The Cancer Genome Atlas (TCGA) (https://www.cancer.gov) and 749 glioma samples and 20 normal brain samples from the Chinese Glioma Genome Atlas (CGGA) (http://www.cgga.org.cn). The samples’ clinical information is shown in Supplementary Table S1, which mainly included glioma grade, 1p19q deletion status, IDH mutation status, age, and survival information.

### Bioinformatics Analysis of TXNDC12

The differences in TXNDC12 expression between normal brain tissue and gliomas were examined by Wilcoxon test. The relationship between TXNDC12 expression and the clinical characteristics of glioma patients was analyzed using the Kruskal test or Wilcoxon test. Survival and ROC curves were plotted using the Survival and ROC Curves package. The scoring of sample immune gene sets was completed using GSVA, Limma, and GSEABase software packages, and an immune heatmap was created using heatmap software. The CIBERSORT R script v1.03 calculated the infiltration of immune cells in glioma samples. The median TXNDC12 expression in samples was used as a threshold to divide samples into high and low expression groups, and the Vioplot software package was used to depict the differences in immune cell infiltration between the two groups.

### Quantitative RT‐PCR

According to the manufacturer’s instructions, the total RNA was extracted by TRIzol reagent (Carlsbad Invitrogen, California, United States). The total RNA concentration was detected using a NanoDrop2000 (Thermo Science^TM^). RNA (1,000 ng) was reverse transcribed into cDNA using a qPCR RT Kit (TOYOBO, Japan). FastStart Universal 96 SYBR Green Master (ROX) (Roche, Germany) was used to detect the relative expression of TXNDC12 and housekeeping gene GAPDH by qRT-PCR. The primer sequences used in our procedure were as follows: TXNDC12, 5-GTC​CTG​CTG​ATT​GTG​AAA​ATG​GC-3 and 5-TGATCCATGTCGAG GGTCAAA-3; GAPDH, 5-AAT​CCC​ATC​ACC​ATC​TTC-3 and 5-AGGC TGTTGTCATACTTC-3′. A non-paired t-test was performed between the two groups, and *p* < 0.05 was considered statistically significant.

### Immunohistochemistry of TXNDC12

We selected 3 normal brain tissues, 3 WHO III gliomas, and 3 WHO IV glioma specimens from the pre-embedded wax blocks, and sliced them into 5 μm thick sections. The section tissue was deparaffinized with xylene, 3% hydrogen peroxide blocked the endogenous peroxidase activity, fetal bovine serum blocked at room temperature, and the primary antibody (4°C, overnight) and secondary antibody (2 h, room temperature) were incubated. Next, samples were visualized by using the diaminobenzidine (DAB) substrate kit. The primary antibody was TXNDC12 (A14403, ABclonal), and the secondary antibody was Goat Anti-Rabbit IgG (S0001, Affinity). Brown staining is a positive feature in cells.

### Enrichment Analysis of TXNDC12

The differential genes that were co-expressed with TXNDC12 in samples (cor > 0.5) were screened by cor.test function in the TCGA and CGGA databases, and the intersection was taken using a Venn diagram. The function of differential genes was analyzed using the R package (clusterProfiler, org.Hs.eg.db, enrichplot, ggplot2).

### Statistical Analysis

R × 64 3.5.2, strawberry-perl-5.30.2.1, and GraphPad Prism7 were used for plotting and statistics. *p* < 0.05 was considered statistically significant.

## Results

### TXNDC12 is Associated With Poor Prognosis of Glioma Patients

**FIGURE 1:**
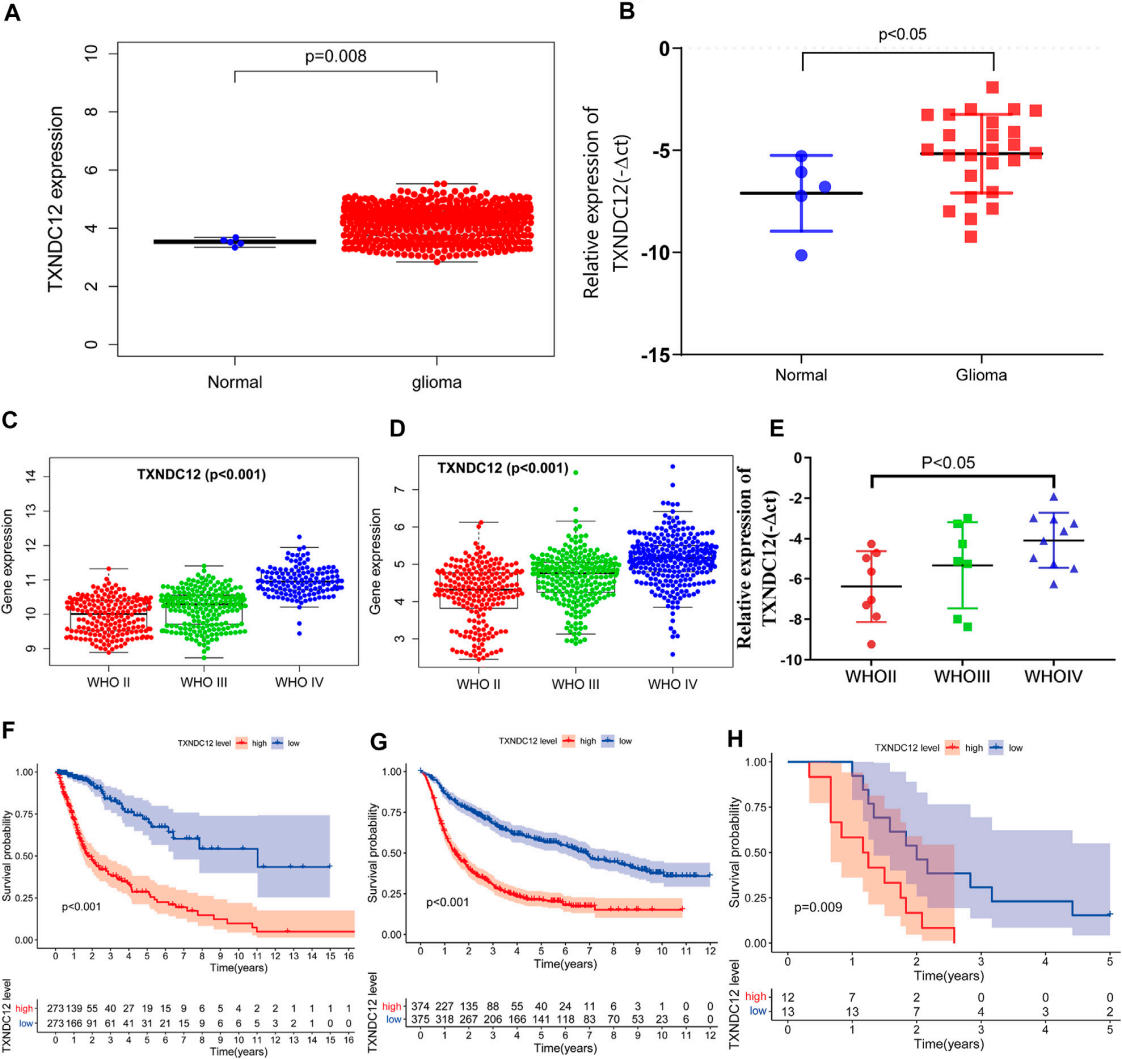
TXNDC12 expression in TCGA **(A)** and surgical resection samples of glioma in our hospital **(B)**. The relationship between TXNDC12 expression and WHO grade of glioma: TCGA **(C)**, CGGA **(D)**, and surgical resection samples of glioma in our hospital **(E)**. Survival curves of glioma patients from TCGA **(F)**, CGGA **(G)**, and our hospital **(H)**.

In samples from TCGA, TXNDC12 expression was significantly higher in glioma tissues compared to normal brain tissues (Figure 1A, *p* < 0.01). We further analyzed the relationship between TXNDC12 expression and glioma pathology grade. We found that TXNDC12 expression increased significantly as glioma grade increased, both in TCGA (Figure 1C, *p* < 0.01) and CGGA (Figure 1D, *p* < 0.01). Survival curve analysis showed that the prognosis of the TXNDC12 high expression group was poorer than that of the low expression group in both TCGA (Figure 1F, *p* < 0.01) or CGGA (Figure 1G, *p* < 0.01) (the cutoff value is the median). All the above results were verified by PCR of clinical glioma samples and the follow-up results of patients in our hospital (Figures 1B,E,H), *p* < 0.05).

### TXNDC12 is an Independent Prognostic Factor for Patients With Glioma

**FIGURE 2:**
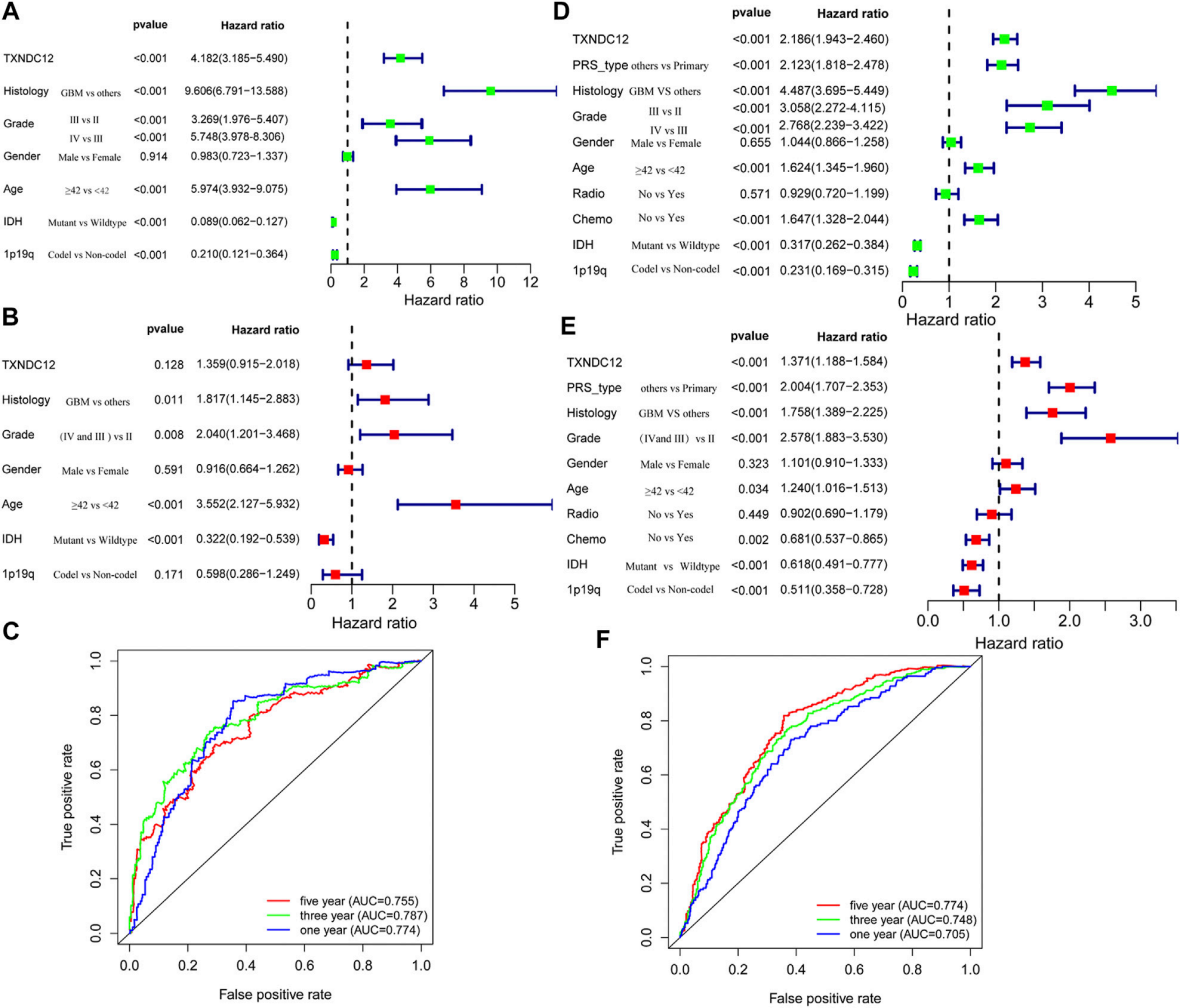
TXNDC12 is an independent prognostic factor for patients with glioma. Univariate analysis, multivariate analysis, and ROC curve analysis of samples from TCGA **(A–C)** and CGGA **(D–F)** database, respectively (Histology refers to the pathological type of glioma; PRS-type refers to primary glioma, recurrent glioma and secondary glioma).

We analyzed the relationship between TXNDC12 and the prognosis of patients with glioma by establishing a Cox regression model. Univariate analysis revealed that TXNDC12 is a significant risk factor for glioma in TCGA-RNA-seq (HR = 4.182, 95% CI = 3.185–5.490, *p* < 0.001) (Figure 2A) and CGGA-RNA-seq (HR = 2.186, 95%CI = 1.943–2.460, *p* < 0.001) (Figure 2D). We found that TXNDC12 is an independent risk factor for glioma in CGGA-RNA-seq by multivariate analysis (HR = 1.371, 95%CI = 1.188–1.584, *p* < 0.001) (Figure 2E). However, the result was not statistically significant in TCGA-RNA-seq (HR = 1.359, 95% CI = 0.915–2.018, *p* = 0.128) (Figure 2B). Furthermore, we found that TXNDC12 was an prognostic factor for 3-year and 5-year survival in glioma patients both in TCGA-RNA-seq (AUC_3year_ = 0.787, AUC_5year_ = 0.755) (Figure 2C) and CGGA-RNA-seq (AUC_3year_ = 0.748, AUC_5year_ = 0.774) (Figure 2F) by ROC curves.

To test the validity and authenticity of the analysis results from TCGA and CGGA databases applicable to actual clinical patients, we collected follow-up data of glioma patients in our hospital (Table 1) for validation. The results showed that expression of TXNDC12 (HR = 1.604, 95%CI = 1.178–2.182, *p* < 0.005) and WHO grade of glioma (HR = 2.059, 95% CI = 1.158–3.662, *p* < 0.05) were risk factors for glioma patients. However, patient age, gender, KPS (Karnofsky Performance Scale) score, and tumor location were not statistically significant.

**TABLE 1:** Association of TXNDC12 expression is with the clinicopathological characteristics of patients with glioma.

Clinical features	Patient number	TXNDC12 expression			Univariate analysis		
		High	Low	HR		95%CI	*p*-value
**TXNDC12**	25	13	12	1.604		(1.178–2.182)	**0.003**
**Age**				>=48 vs < 48			
<48	12	6	6	reference group			
>=48	13	7	6	0.82		(0.36–1.86)	0.63
**Gender**				Male vs Female			
Female	11	5	6	reference group			
Male	14	8	6	2.15		(0.92–5.05)	0.08
**WHO grade**				(Ⅳ or Ⅲ) vs Ⅱ			
Ⅱ	8	3	5	reference group			
Ⅲ	7	3	4	1.146		(0.379–3.465)	0.809
Ⅳ	10	7	3	2.059		(1.158–3.662)	**0.014**
**KPS score**				≥80 vs < 80			
<80	9	4	5	reference group			
≥80	16	9	7	1.49		(0.62–3.55)	0.37
**Tumor location**				(Frontal, Parietal or Temporal lobe) vs Occipital lobe)			
Occipital lobe	3	2	1	reference group			
Parietal lobe	6	2	4	1.281		(0.571–2.874)	0.548
Temporal lobe	5	3	2	1.614		(0.307–8.473)	0.571
Frontal lobe	11	6	5	1.582		(0.345–7.264)	0.555
**Tumor volume(cm3)**				≥37 vs <37			
<37	11	5	6	reference group			
≥37	14	8	6	0.937		(0.409–2.147)	0.878
**Postsurgical radiotherapy**				No vs Yes			
Yes	18	11	7	reference group			
No	7	2	5	0.502		(0.198–1.272)	0.146
**Postsurgical TMZ therapy**				No vs Yes			
Yes	16	8	8	reference group			
No	9	5	4	0.672		(0.279–1.620)	0.376

KPS score, Karnofsky performance score; HR, Hazard Ratio; CI, confidence interval; bold values represent statistical significance; *p* < 0.05 considered statistically significant.

### The Relationship Between TXNDC12 and 1p19q Status

**FIGURE 3:**
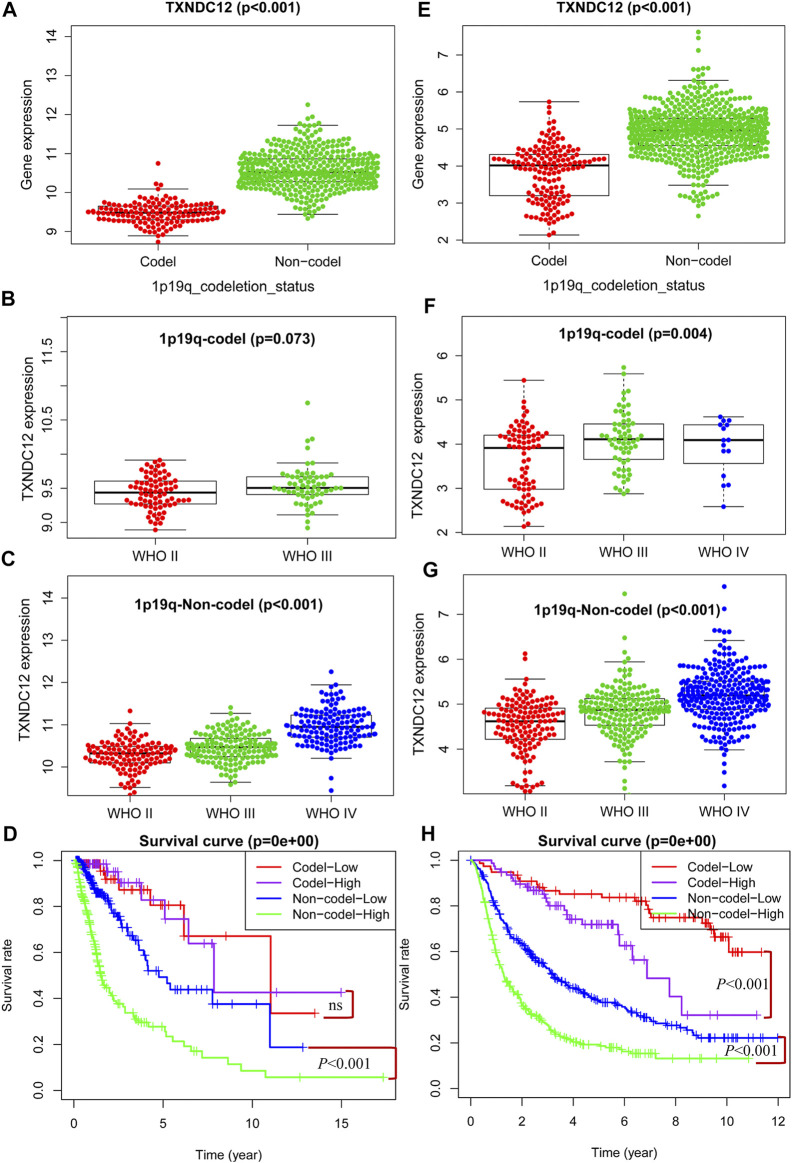
Combined detection of 1p19q and TXNDC12 is meaningful for prognostic judgment of glioma. The difference of TXNDC12 expression in different 1p19q status gliomas from TCGA **(A)** and CGGA **(E)**. The relationship between TXNDC12 expression and different glioma grade in 1p19q Codel glioma from TCGA **(B)** and CGGA **(F)**. The relationship between TXNDC12 expression in 1p19q Non-codel glioma and glioma grade from TCGA **(C)** and CGGA **(G)**; Survival curve for combined test of TXNDC12 and 1p19q from TCGA **(D)** and CGGA **(H)**.

1p19q has important prognostic implications for glioma patients as a molecular marker for pathological classification. We have demonstrated that high expression of TXNDC12 in glioma predicts adverse outcomes in patients. To explore whether there is any relationship between TXNDC12 and 1p19q status, we found that the expression of TXNDC12 was significantly lower in the 1p19q Codel (1p19q co-deletion) group compared to the 1p19q Non-codel (1p19q non-codeletion) group, both in TCGA (Figure 3A, *p* < 0.005) and CGGA (Figure 3E, *p* < 0.005). The expression of TXNDC12 in the 1p19q codel subgroup increased with glioma WHO grade (Figure 3F, *p* < 0.005) in CGGA. However, this difference was not statistically significant in TCGA (Figure 3B, *p* = 0.073). In the 1p19q Non-codel subgroup, TXNDC12 expression significantly increased with the rise of the grade of gliomas in both TCGA (Figure 3C, *p* < 0.005) and CGGA (Figure 3G, *p* < 0.005). In the 1p19q Non-codel subtype, TCGA (Figure 3D, *p* < 0.005) and CGGA (Figure 3H, *p* < 0.005), patients in the TXNDC12 high expression group had a worse prognosis than those in the TXNDC12 low expression group. In the 1p19q Codeletion subtype, CGGA (Figure 3H, *p* < 0.005),patients in the TXNDC12 high expression group had a worse prognosis than those in the TXNDC12 low expression group, while this change was not statistically significant in the TCGA database.

### The Relationship Between TXNDC12 and Isocitrate Dehydrogenase Status

**FIGURE 4:**
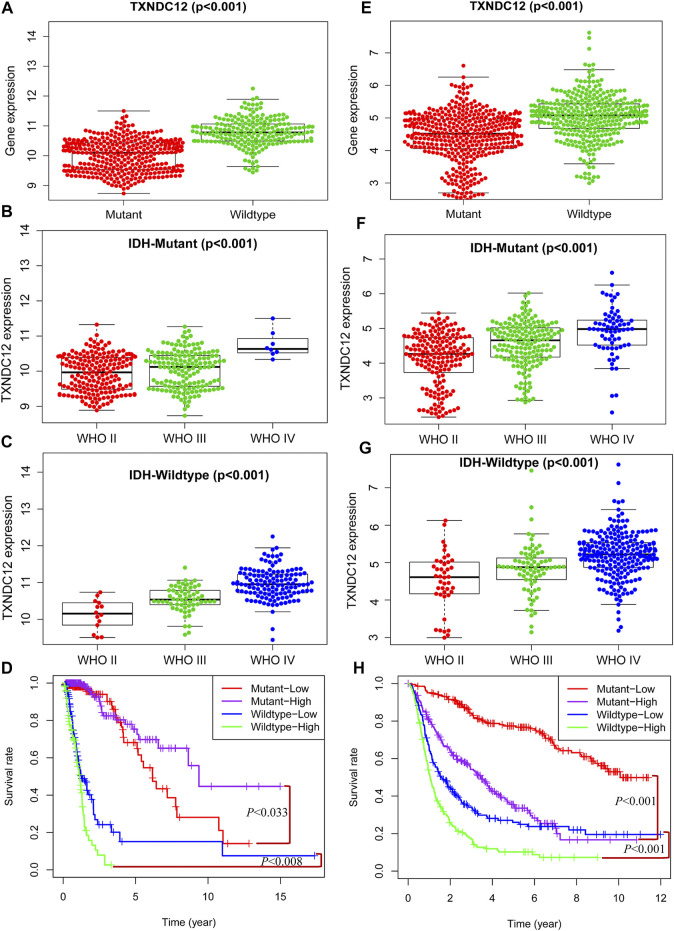
Combined detection of IDH and TXNDC12 is meaningful for prognostic judgment of glioma. The difference of TXNDC12 expression in different IDH status gliomas from TCGA **(A)** and CGGA **(E)**. The relationship between TXNDC12 expression and different glioma grade in IDH Mutant glioma from TCGA **(B)** and CGGA **(F)**. The relationship between TXNDC12 expression in IDH Wildtype glioma and glioma grade from TCGA **(C)** and CGGA **(G)**; Survival curve for combined test of TXNDC12 and IDH status from TCGA **(D)** and CGGA **(H)**.

Similar to 1p19q, IDH is another glioma typing biomarker widely used clinically. We found that the expression of TXNDC12 in the IDH Wildtype group was significantly higher than that in the IDH Mutant group in TCGA (Figure 4A, *p* < 0.005) and CGGA (Figure 4E, *p* < 0.005). In the IDH Mutant subgroups, TCGA (Figure 4B, *p* < 0.005) and CGGA (Figure 4F, *p* < 0.005), the expression of TXNDC12 increased significantly with the rise of glioma grade, and the same results were observed in the IDH Wildtype subgroup (Figure 4C, *p* < 0.005 and Figure 4G, *p* < 0.005). In the IDH Wildtype subtype, TCGA (Figure 4D, *p* < 0.005) and CGGA (Figure 4H, *p* < 0.005), patients in the TXNDC12 high expression group had a worse prognosis than those in the TXNDC12 low expression group. In the IDH Mutant subtype, CGGA (Figure 4H, *p* < 0.005), patients in the TXNDC12 high expression group had a worse prognosis than those in the TXNDC12 low expression group. However, in the TCGA, the prognosis of MT-high (IDH Mutant combined with TXNDC12 high expression) is better than that of MT-low (IDH Mutant combined with TXNDC12 low expression).

### The Effect of TXNDC12 Combined With IDH and 1p19q on Glioma

**FIGURE 5:**
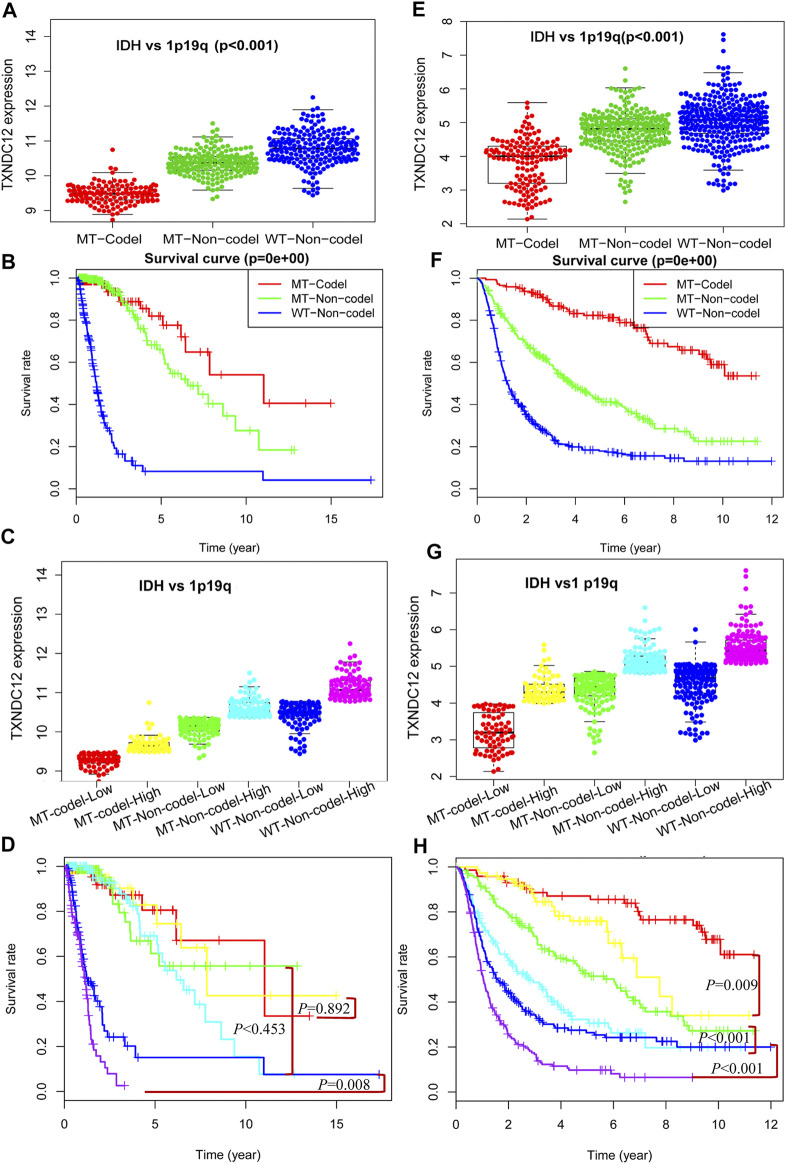
Comprehensive analysis of TXNDC12 combined with 1p19q and IDH status on the prognosis of glioma. Detection of TXNDC12 expression, 1p19q status and IDH status in samples from TCGA **(A)** and CGGA **(E)**. Survival curve of patients with three subtypes of glioma: TCGA **(B)** and CGGA **(F)**; Each type of part **(A)** and **(E)** is divided into two subgroups in this figure (division cut point value is the median of TXNDC12 expression level). Part **(C)** corresponds to part **(A)**, part **(G)** corresponds to part **(E)**. Survival curves of patients with six subtypes of glioma samples from TCGA **(D)** and CGGA **(H)**: The subtypes represented by different colors have the same meaning as those represented in parts **(C)** and **(E)**.

In previous studies, we have proved the significance of TXNDC12 alone, TXNDC12 combined with IDH, and TXNDC12 combined with 1p19q on glioma patient prognosis. However, we know that these three molecular changes may occur in the same patient in actual clinical cases. Reliant on any single molecule to analyze glioma patients’ pathological condition and prognosis may be relatively weak and confusing. Therefore, we combined IDH, 1p19q, and TXNDC12 to explore their combined effects on patient prognosis with glioma. In Figure 5A (TCGA, *p* < 0.005) and Figure 5E (CGGA, *p* < 0.005), we found that TXNDC12 had the lowest expression in the MT-Codel (IDH Mutation combining 1p19q Codeletion) group, while the MT-Non-codel (IDH Mutant combining 1p19q Non-codeletion) was moderate, and the WT-Non-codel (IDH Wildtype combining 1p19q Non-codeletion) had the highest expression. Survival analysis showed that the MT-Codel subgroup had the longest survival time, followed by the MT-Non-codel group. The WT-Non-codel group had the shortest survival time (Figure 5B, *p* < 0.005; and Figure 5F, CGGA, *p* < 0.005). To further investigate the effect of the difference in TXNDC12 expression among the three subtypes on glioma prognosis, we divided the samples of each subtype into TXNDC12 high-expression and low-expression groups according to the median TXNDC12 expression in the samples. That is, the above three groups were divided into six subgroups (Figure 5C, TCGA; and Figure 5G, CGGA), and survival curves were drawn for Figure 5D (TCGA) and Figure 5H (CGGA). The results showed that TXNDC12 high expression group had shorter survival within the same subtype compared to the low expression group in CGGA (Figure 5D). The WT-Non-codel subtype also showed the same results in TCGA, but the difference in survival curves was not statistically significant between MT-codel and MT-Non-codel subtypes (Figure 5H).

### The Relationship Between TXNDC12 and Immune Infiltration in Glioma

**FIGURE 6:**
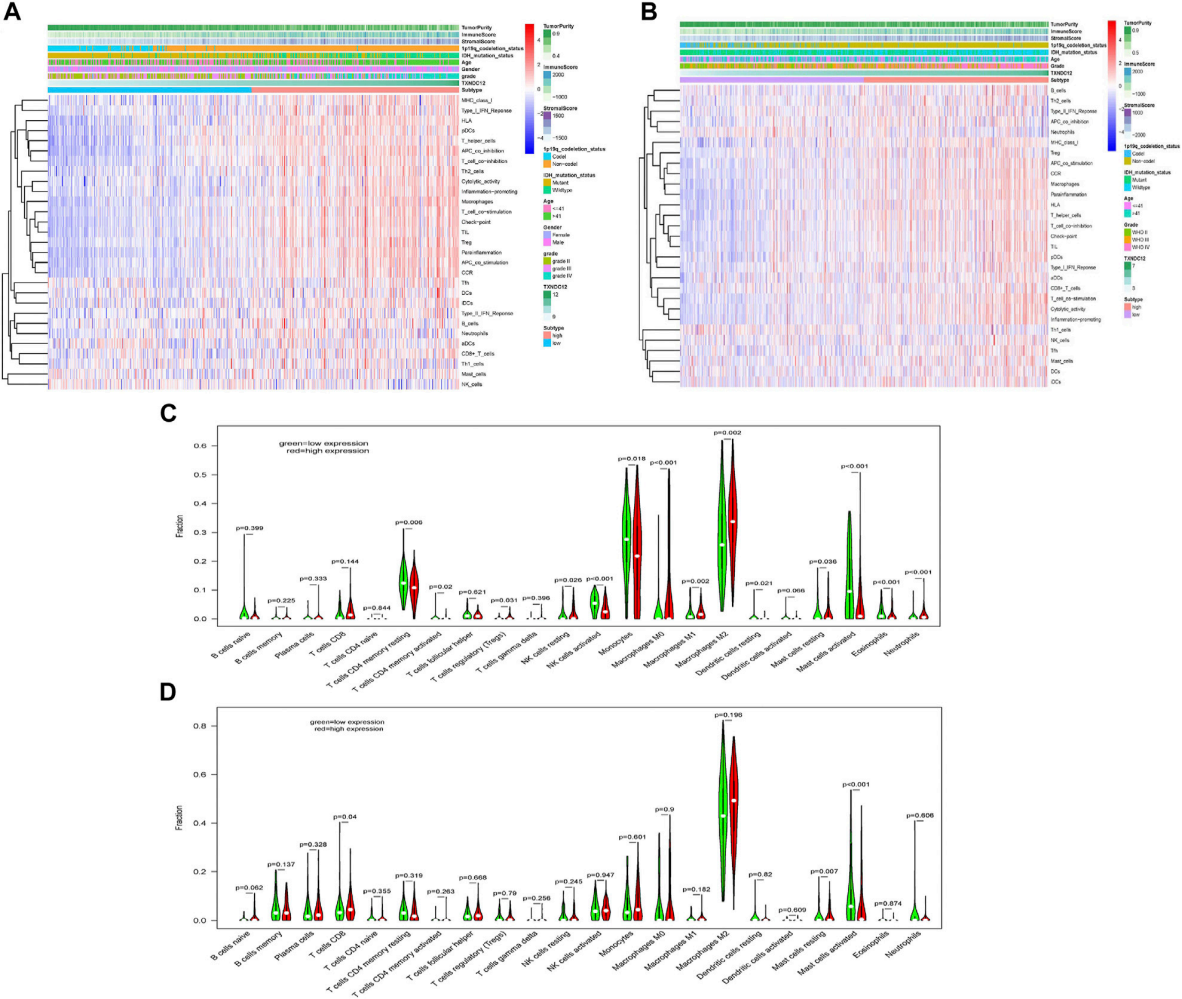
The effect of TXNDC12 on the immune microenvironment of glioma. In TCGA **(A)** CGGA **(B)**, heatmap of the patient’s clinical characteristics and tumor local immune status. The infiltration of immune cells in the TXNDC12 high and low expression subgroups of glioma samples from TCGA **(C)** and CGGA **(D)**.

We know that there is an immunosuppressive phenomenon in glioma lesions, which affects the efficacy of many therapeutic measures against glioma. To investigate the relationship between TXNDC12 and the local immune microenvironment glioma, we analyzed data from TCGA (Figure 6A) and CGGA (Figure 6B) databases. The results showed that the clinical features of gliomas in the TXNDC12 high expression group were mainly associated with IDH wild-type, 1p19q non-codeletion, age >41 years, higher WHO grade, reduced tumor cell purity, increased immune cells, and extracellular matrix in the tumor tissue. Further analysis of the infiltrating immune cell components revealed that infiltration of immune cells in glioma tissue is inconsistent across populations and mast cells decreased in the TXNDC12 high expression group (Figures 6C,D).

### The Protein Expression Level of TXNDC12 in Glioma

**FIGURE 7:**
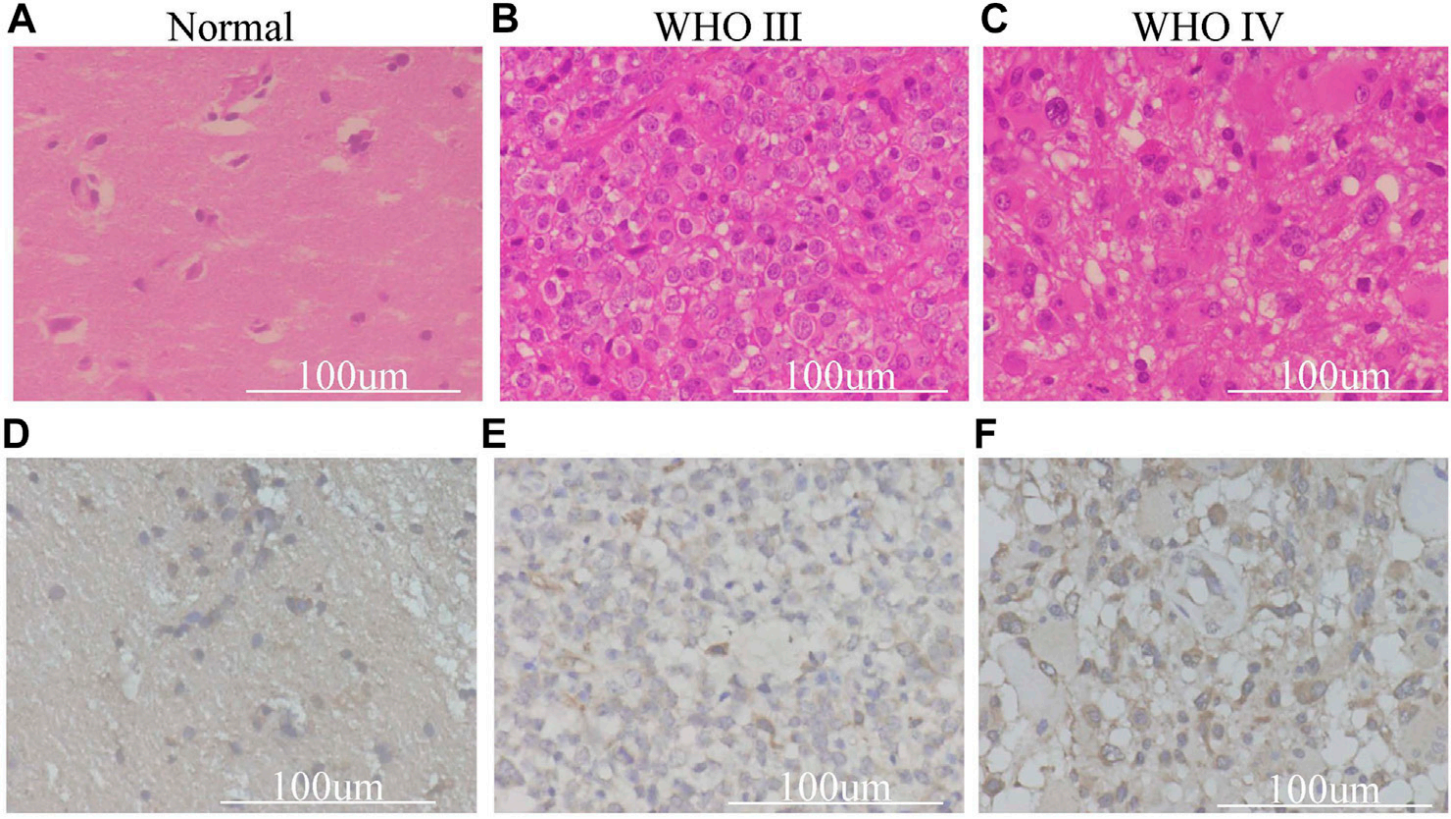
HE staining **(A–C)** and Immunohistochemical **(D–F)** section of surgical samples in our hospital.

We found that the combination of IDH and 1p19q status has an absolute predictive value for the prognosis of glioma patients. The expression of TXNDC12 is significantly increased at the mRNA level in gliomas; however, it is unclear whether the expression of the protein encoded by the final target TXNDC12 gene also has corresponding changes. We took normal brain tissue, WHO III, and WHO IV glioma tissues for HE staining (Figures 7A–C) and performed TXNDC12 immunohistochemical analysis (Figures 7D–F,). HE staining showed that WHO III and WHO IV gliomas were dense, with large, and dark stained nuclei and irregular shape (Figures 7B,C). Immunohistochemistry showed an increase in TXNDC12-positive cells in WHO III and WHO IV glioma tissues compared to normal brain tissues (Figure 7E,F).

### Functional Enrichment Analysis of Genes Related to TXNDC12 and Glioma Staging

**FIGURE 8:**
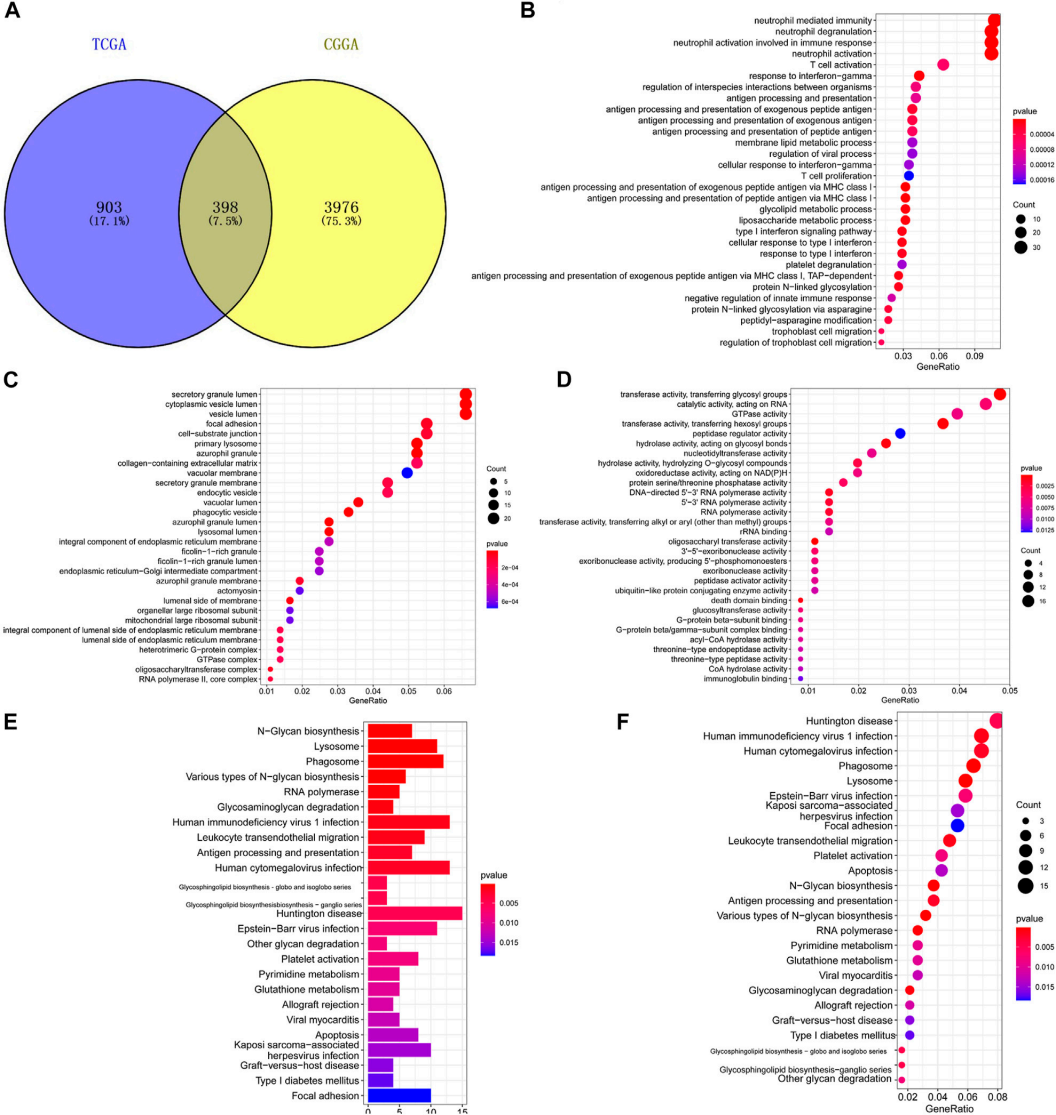
Functional enrichment analysis of genes related to TXNDC12 and glioma staging. Intersection of TXNDC12 Co-Expressed Genes in TCGA and CGGA **(A)**. Biological processes associated with the co-expressed gene of TXNDC12 **(B)**. Cellular components associated with the co-expressed gene of TXNDC12 **(C)**. Molecular functions associated with the co-expressed gene of TXNDC12 **(D)**. KEGG analysis for genes co-expressed with TXNDC12 **(E, F)**.

Co-expression analysis yielded 1301 TXNDC12-related genes in TCGA and 4,374 in CGGA and intersected these genes to obtain 398 genes, as shown in Figure 8A. The GO enrichment analysis of the biological processes (Figure 8B), cellular components (Figure 8C), and molecular functions (Figure 8D) involved in these genes revealed that the functions of these genes were mainly focused on RNA synthesis, antigen presentation and processing, neutrophil-mediated immune response, and glycosylation. The KEGG analysis (Figures 8E,F) shows that major pathways are enriched in glycosyl transfer reaction and DNA transcription.

## Discussion

Glioma is the most common primary intracranial tumor, accounting for 75% of malignant brain tumors[9]. Although it has lower morbidity compared to tumors in other systems throughout the body, it causes a high mortality rate[22]. Among them, glioblastoma has the lowest OS rate, with only 4.7% of patients surviving within 5 years after diagnosis[23]. In recent years, emerging therapeutic modalities such as immunotherapy and Tumor Treating Fields have been attempted to be used as treatments for gliomas, however, no breakthrough progress has been shown[24–26]. Tumors are caused by abnormal tissue cell changes at the genetic level, and gliomas are no exception[27]. Many complex pathways are involved, and the level of awareness of glioma limits the ability to diagnose and treat tumors[28,29]. Genetic therapy is a significant breakthrough in solving the problem of tumor treatment. The development of sequencing technology and bioinformatics has led to the discovery of many specific biomarkers, which are of great therapeutic value[8,30].

We screened the TXNDC12 gene using public databases and found that it is highly expressed in gliomas, and the expression level increased significantly with the level of gliomas. The PCR results of glioma samples from our hospital verified this conclusion. Further regression analysis found that TXNDC12 is an independent risk factor for the prognosis of glioma, which has a certain clinical application value. However, in the multivariate risk analysis for TXNDC12, it was found that the analysis results for the TCGA database (HR = 1.359, 95% CI = 0.915–2.018, *p* = 0.128) were not statistically significant. We found that this may be related to different races and sample sizes.

For many years, the pathological classification and grading of gliomas have been based on the morphological criteria of microscopic tumor tissues[30]. However, in clinical practice, it has been found that this standard does not accurately reflect the actual course of glioma and has a limited guiding value for the treatment and prognosis of patients[31]. With the discovery of the significance of molecular changes such as IDH and 1p19q in gliomas, the WHO 2016 version of the pathological classification of gliomas introduced these standards[32,33]. We combined IDH and 1p19q for analysis and found that the expression level of TXNDC12 in IDH mutant type was significantly lower than that in IDH wild-type, and the expression level of the 1p19q deletion type was significantly lower than that of the 1p19q non-deletion type. The TXNDC12 low expression group of different subtypes had better prognosis, and the combination of these three subtypes can have a better guiding significance for the pathological classification and prognosis judgment of glioma.

In addition to tumor cells, various extracellular matrices, cytokines, and infiltrating immune cells of various types are components that together make up the glioma tumor microenvironment[34]. There is still much work to investigate the role of these immune cells in the microenvironment of glioma. However, an increasing number of researchers are discovering that the non-tumor cell components in glioma also play an important role in the occurrence and progression of glioma[35]. Many relevant research results are currently being reported. For example, tumor-associated macrophages in the tumor microenvironment are involved in forming the local immunosuppressive state in glioma[36], which explains the unsatisfactory efficacy of various current immunotherapeutic measures against glioma[37,38]. These results indicate that glioma’s local immune microenvironment plays a vital role in treating glioma[39]. Therefore, we also analyzed the infiltration status of glioma immune cells in the samples. The results showed that in the group with high TXNDC12 expression, tumor cell purity in tumor tissues decreased, immune cells, and extracellular matrix increased, and mast cell activity decreased. Some researchers found that glioma tissues induce migration of mast cells to glioma tissues by producing cytokines such as glioma-derived macrophage mobility inhibitory factor[40]. These mast cells reduce the proliferative and invasive capacity of glioma cells and induce their differentiation by depleting the potency of glioma stem cells, possibly by inactivating the STAT3-mediated pathway through GSK3β downregulation[41]. Based on the above results, we infer that mast cells may be involved in the generation of poor prognosis in patients with TXNDC12 high-expressing glioma.

We found that TXNDC12 is involved in the poor prognosis of glioma. To further investigate the mechanism by which TXNDC12 is involved in the malignant prognosis of glioma, we analyzed the expression of TXNDC12 protein in glioma and normal brain tissue and found that TXNDC12 protein expression was significantly increased in gliomas and appeared to be positively correlated with glioma grade. Unfortunately, due to the small number of clinical glioma samples we collected for immunohistochemical analysis (especially for grade II gliomas), we were unable to perform a valid statistical analysis of the differences in TXNDC12 expression in different gliomas as well as in normal brain tissue, which is a drawback of this study. Therefore, we hypothesized that TXNDC12 is involved in the malignant progression of gliomas through the expression of corresponding functional proteins. In addition, functional enrichment analysis of TXNDC12 functionally related genes revealed that the functions of these genes are mainly focused on the regulation of the activities of various glycosyltransferases such as glucosyltransferase and oligosaccharyltransferase, as well as in the process of RNA synthesis. The role of glycosyltransferases in glioma has also been extensively studied in several studies that have found glycosyltransferases to be associated with the development and aggressive growth of various malignant tumors such as breast cancer, glioblastomas, and glioma[42–44]. A small molecule inhibitor of N-acetylglucosaminyltransferase (MGAT5)[45], PST3.1a, can significantly inhibit the invasive and proliferative capacity of glioma by inhibiting the signaling of βR and FAK-related pathways, and in combination with TMZ can significantly prolong the survival of glioma patients[46]. Therefore, we speculate that TXNDC12 is likely to confer enhanced proliferation and invasiveness to this type of glioma by increasing the activity of related glycosyltransferases. If our hypothesis is confirmed, the development of new drugs targeting this glycosyltransferase enzyme is expected to bring new solutions to glioma drug therapy and provide an effective complement to current temozolomide therapy, which is plagued by widespread drug resistance problems. However, as one of the shortcomings of this experiment, we did not design a validation experiment to confirm our conjecture.

## Conclusion

We analyzed the vital role of the TXNDC12 gene in glioma and showed that TXNDC12 is highly expressed in glioma tissue and that it is significantly related to pathological grade and poor prognosis. So, TXNDC12 may serve as a potential molecular marker for glioma pathological grade and prognosis.

## Data Availability

The raw data supporting the conclusions of this article will be made available by the authors, without undue reservation.
