# Supplementary material for: Clinical Value of TXNDC12 Combined With IDH and 1p19q as Biomarkers for Prognosis of Glioma
Source: Pathol Oncol Res. 2021 Sep 22;27:1609825. doi: 10.3389/pore.2021.1609825 (PMC8493877; doi:10.3389/pore.2021.1609825)
Supplement: Supplementary file 1 [file Table1.docx]

| **Supplementary Table 1: Characteristics of patients with glioma based on TCGA and CGGA.** | | | | | |
| --- | --- | --- | --- | --- | --- |
| **Characteristics** | | **TCGA** | | **CGGA** | |
|  |  | **Number of cases** | **Percentages**  **(%)** | **Number of cases** | **Percentages**  **(%)** |
| **Gender** | Female | 231 | 42.31% | 307 | 40.99% |
|  | male | 315 | 57.69% | 442 | 59.01% |
| **Age** | ≤42 | 218 | 39.93% | 370 | 49.40% |
|  | ＞42 | 328 | 60.07% | 379 | 50.60% |
| **IDH_status** | Mutant | 342 | 62.64% | 410 | 54.74% |
|  | Wildtype | 204 | 37.36% | 339 | 45.26% |
| **1p19q_status** | Codel | 138 | 25.27% | 155 | 20.69% |
|  | Non-codel | 408 | 74.73% | 594 | 79.31% |
| **Grade** | Ⅱ | 194 | 35.53% | 218 | 29.11% |
|  | Ⅲ | 215 | 39.38% | 240 | 32.04% |
|  | Ⅳ | 137 | 25.09% | 291 | 38.85% |
| **Histology** | A* | 46 | 8.42% | 75 | 10.01% |
|  | AA | 104 | 19.05% | 75 | 10.01% |
|  | AO | 77 | 14.10% | 37 | 4.94% |
|  | AOA | 34 | 6.23% | 128 | 17.09% |
|  | O | 107 | 19.60% | 39 | 5.21% |
|  | OA | 41 | 7.51% | 104 | 13.89% |
|  | GBM | 137 | 25.09% | 291 | 38.85% |
| **PRS_type** | Primary |  |  | 502 | 67.02% |
|  | Recurrent |  |  | 222 | 29.64% |
|  | Secondary |  |  | 25 | 3.34% |
| **Radio_status** | No |  |  | 124 | 16.56% |
|  | Yes |  |  | 625 | 83.44% |
| **Chemo_status** | No |  |  | 229 | 30.57% |
|  | Yes |  |  | 520 | 69.43% |

***A: Astroglioma; AA: Anaplastic astrocytoma; AO: Anaplastic oligodendroglioma**

**AOA: Anaplastic oligoastrocytoma; O: Oligodendroglioma; OA: Oligoastrocytoma**

**GBM: Glioblastoma**
